# Supplementary material for: High-Performance Graphene Nanowalls/Si Self-Powered Photodetectors with HfO2 as an Interfacial Layer
Source: Nanomaterials (Basel). 2023 May 19;13(10):1681. doi: 10.3390/nano13101681 (PMC10222753; doi:10.3390/nano13101681)
Supplement: Supplementary file 1 [file nanomaterials-13-01681-s001.zip › nanomaterials-2405140-supplementary.pdf]

# Supporting Information

# High Performance Graphene Nanowalls/Si Self-powered Photodetectors with $HfO_2$ as an Interfacial Layer

Yuheng Shen[1,2], Yulin Li[1,2], Wencheng Chen[1,2], Sijie Jiang[1], Cheng Li[1] and Qijin Cheng[1, 2,*]

1) School of Electronic Science and Engineering, Xiamen University, Xiamen, 361102, P. R. China.

2) Shenzhen Research Institute of Xiamen University, Shenzhen, 518000, P. R. China.

*Correspondence: E-mail: qijin.cheng@xmu.edu.cn

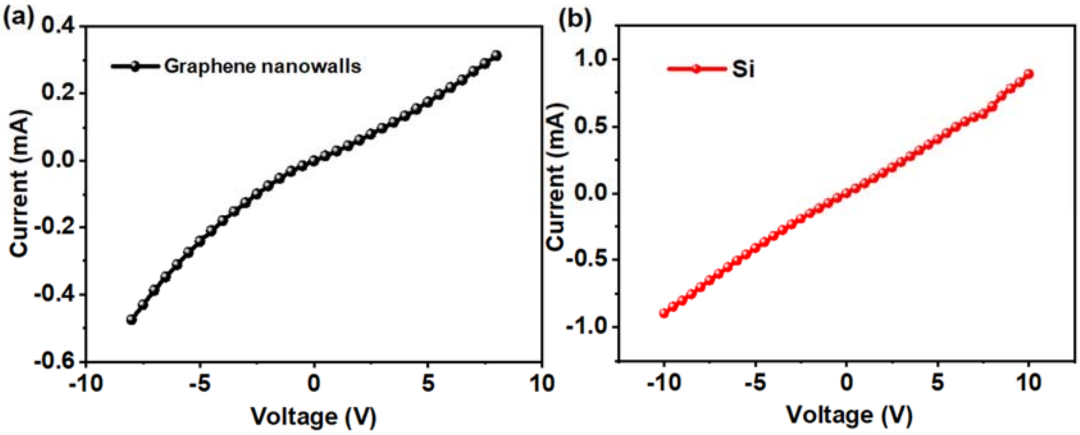


**Figure S1**. Ohmic contact of **(a)** GNWs and **(b)** Si.

The current-voltage characteristic curves for Cr/Au-GNWs-Cr/Au and Cr/Au-Si-Cr/Au are shown in Figure S1a and S1b, respectively. The almost linear I-V characteristic curves indicate that quasi ohmic contact is formed.

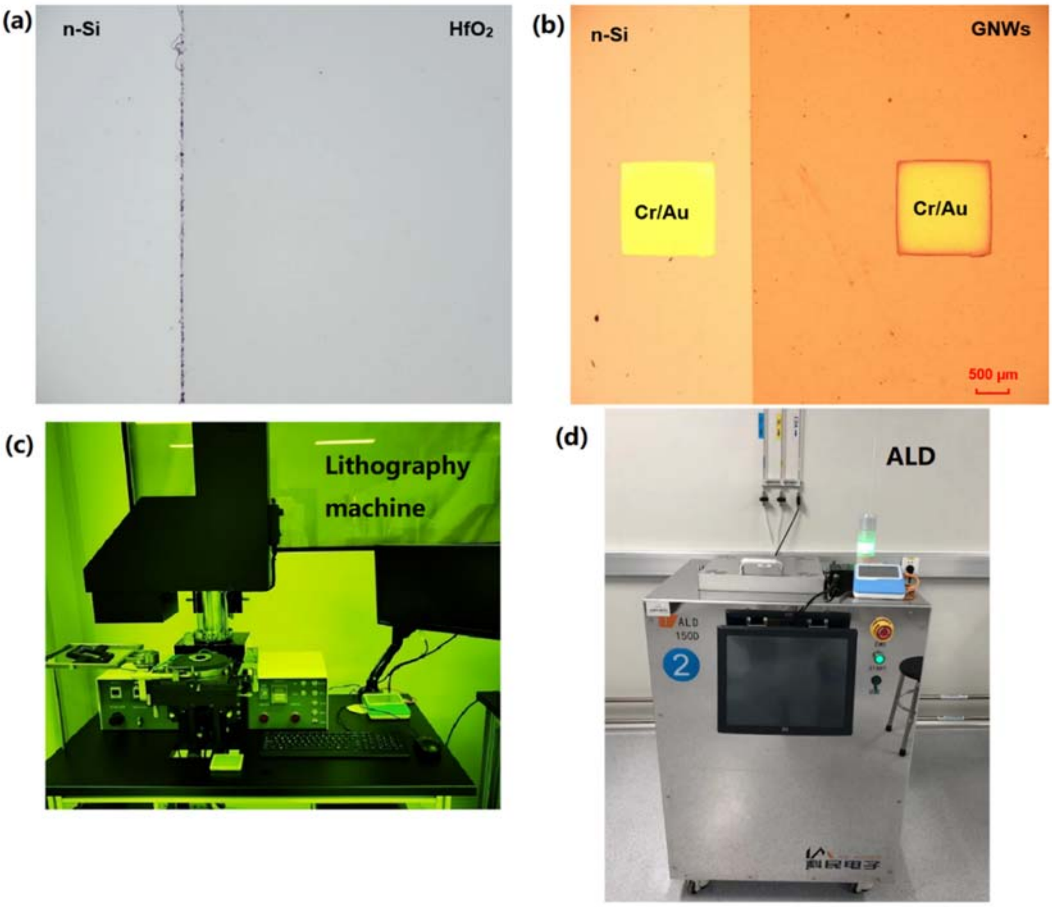


**Figure S2. (a)** Photograph of the Si substrate with a 3 nm $HfO_2$ layer (the two-thirds area of the Si substrate was covered with a 3 nm $HfO_2$ layer). **(b)** Photograph of the GNWs/$HfO_2$/Si photodetector. **(c)** Photograph of lithography equipment. **(d)** Photograph of ALD equipment.

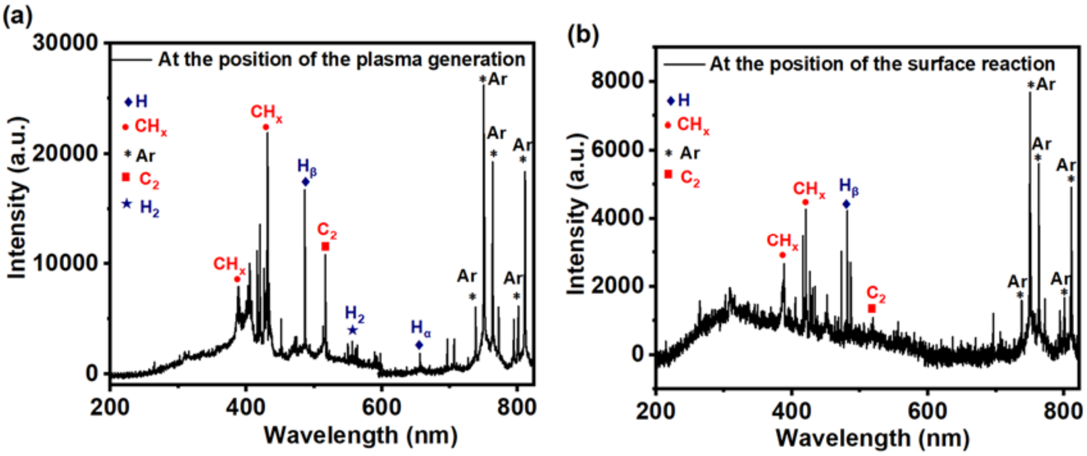


**Figure S3.** OES spectra at different positions of the remote PECVD system: **(a)** at the

position of the plasma generation and **(b)** at the position of the surface reaction.

Figure S3 shows OES spectra at different positions of the remote PECVD system in the growth process of GNWs under the condition of a $CH_4$/Ar flow rate ratio of 10 sccm/40 sccm, an RF power of 300 W, a growth temperature of 900 °C and a growth time of 120 s. As shown in Figure S3a, a series of spectral lines, including $C_2$, $CH_x$, Ar, $H_\alpha$, $H_\beta$, $H_2$, etc., can be observed at the position of the plasma generation. Notably, Figure S3a exhibits several argon spectral lines with a high intensity, located in the 750-820 nm range. In contrast, Figure S3b does not show the spectra lines of $H_\alpha$ and $H_2$ at the position of the surface reaction, which is favorable for the decrease of the etching effect by H-related radicals. Moreover, the intensity of argon spectral lines decreases at the position of the surface reaction, which can effectively reduce the impact of the unwanted ion bombardment.

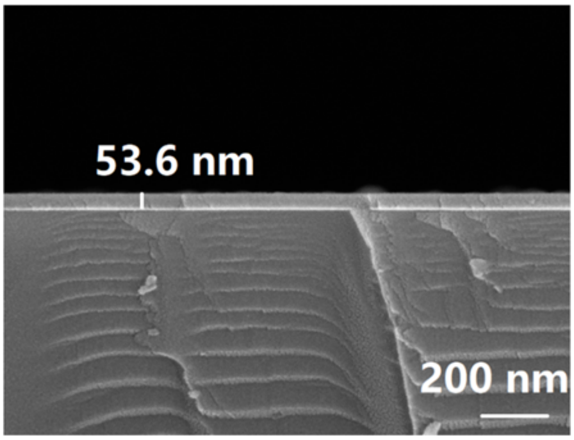


**Figure S4.** A cross-sectional SEM image of $HfO_2$ grown on the Si substrate for 500 cycles.

As shown in Figure S4, the thickness of $HfO_2$ is approximately 53.6 nm and the growth rate is calculated to be 1.07 Å/cycle.

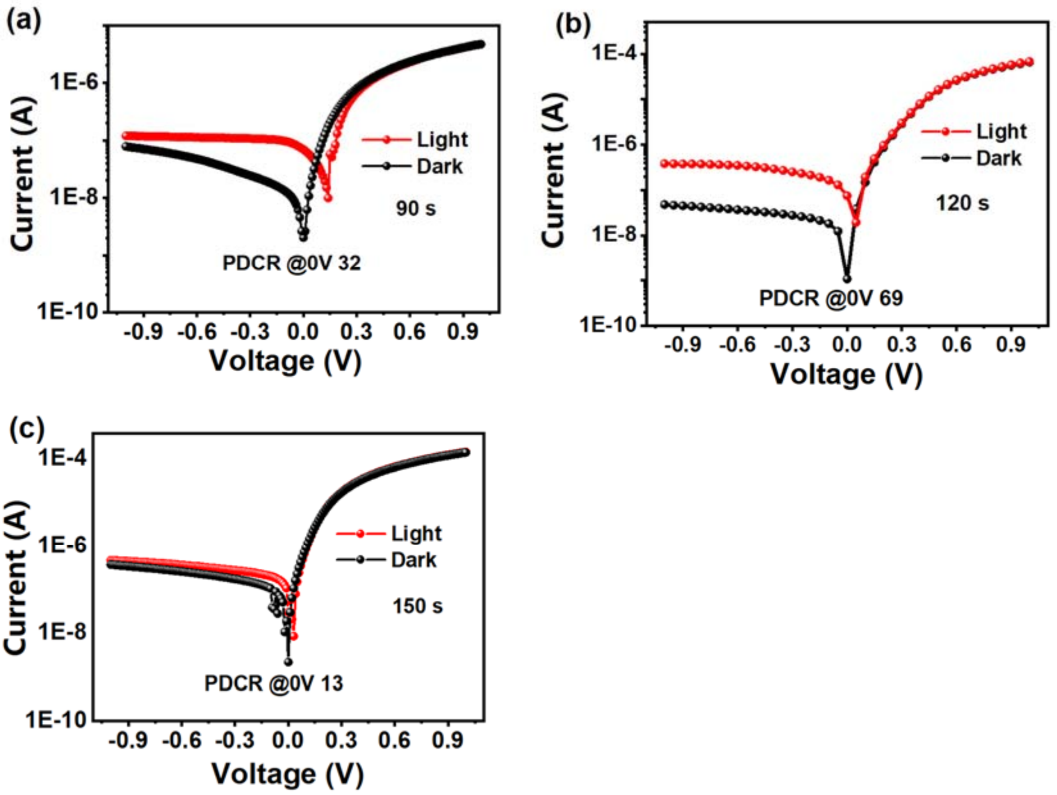


**Figure S5.** I-V characteristic curves of the GNWs/$HfO_2$/Si photodetectors with different growth times of GNWs **(a)** 90 s, **(b)** 120 s, and **(c)** 150 s, respectively.

Figure S5 shows the I-V characteristic curves of the GNWs/$HfO_2$/Si photodetectors with different growth times of GNWs. Table S1 lists the photoelectric parameters of the GNWs/$HfO_2$/Si photodetectors with different growth times of GNWs. As shown in Table S1, the photodetector for GNWs grown for 120 s shows the best performance among all the fabricated devices.

**Table S1.** Photoelectric parameters of the GNWs/$HfO_2$/Si photodetectors with different growth times of GNWs.

| **Time (s)** | **Current under dark condition (A)** | **Current under light condition (A)** | **PDCR @ 0V** | **Responsivity (A/W)** |
|---|---|---|---|---|
| 90 | $2.02 \times 10^{-9}\,A$ | $6.91 \times 10^{-8}\,A$ | 32 | 0.054 |
| 120 | $1.07 \times 10^{-9}\,A$ | $7.38 \times 10^{-8}\,A$ | 69 | 0.058 |
| 150 | $2.16 \times 10^{-9}\,A$ | $2.98 \times 10^{-8}\,A$ | 13 | 0.023 |

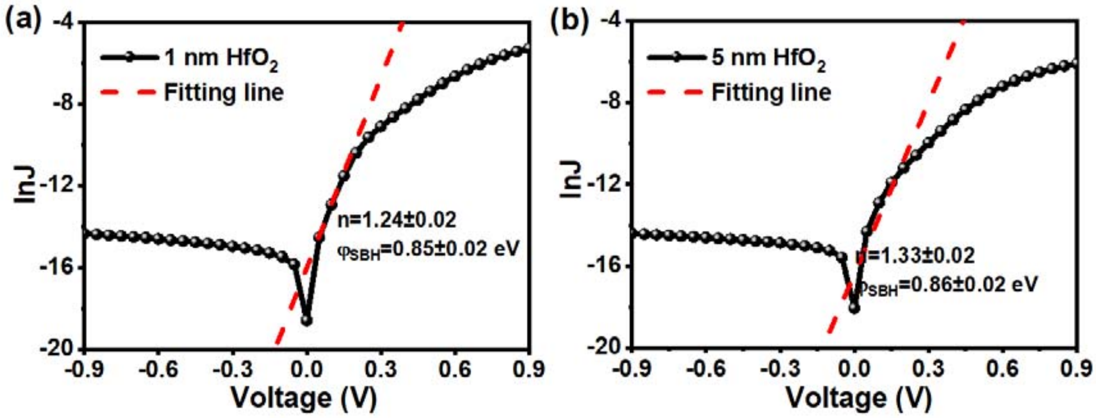


**Figure S6**. lnJ-V curves of the GNWs/Si photodetectors with the thickness of **(a)** 1 nm and **(b)** 5 nm $HfO_2$ layer.

Figure S6 shows the lnJ-V curves of the GNWs/Si photodetectors with different thicknesses of $HfO_2$ interface layer. We can obtain n and $\varphi_{SBH}$ by fitting the linear part of the curve based on the equations (5) and (6). The results of the barrier height for photodetectors with 1 and 5 nm $HfO_2$ are 0.85±0.02 and 0.86±0.02 eV, respectively. The results of the ideality factor for photodetectors with 1 and 5 nm $HfO_2$ are 1.24 and 1.33, respectively.
